# The Diagnostic Challenges in Carotid Cavernous Fistula: A Case Series

**DOI:** 10.7759/cureus.19696

**Published:** 2021-11-18

**Authors:** Krishnadevi Thiyagarajam, Mei Fong Chong, Safinaz Mohd Khialdin

**Affiliations:** 1 Ophthalmology, Kementerian Kesihatan Malaysia (KKM), Kuala Lumpur, MYS; 2 Ophthalmology, Hospital Raja Permaisuri Bainun, Ipoh, MYS; 3 Ophthalmology, Hospital Universiti Kebangsaan Malaysia, Kuala Lumpur, MYS

**Keywords:** ophthalmological findings, carotid-cavernous sinus fistula, proptosis, swollen eye, red eye

## Abstract

A carotid-cavernous fistula (CCF) is an arteriovenous fistula with an abnormal connection between the carotid artery and cavernous sinus that can be sight and life-threatening. The conjunctival injection is often the most prominent feature, and patients are commonly misdiagnosed for other ocular conditions leading to a delay in diagnosis and treatment. All three patients in this case series presented with persistent red eyes. They were all treated for conjunctivitis and only referred for further workup when other progressing ocular symptoms occurred. The diagnosis of CCF was confirmed with digital subtraction angiography and with successful endovascular embolization, their ocular symptoms resolved with preserved optic nerve function. A high index of suspicion in patients presenting with an atypical red eye is very crucial for timely diagnosis of CCF.

## Introduction

A carotid-cavernous fistula (CCF) is an abnormal arteriovenous connection between the cavernous sinus (CS) and internal carotid artery, which can be a direct or indirect fistula. Patients with CCF often present with nonspecific ocular symptoms and signs that can be challenging in CCF diagnosis. This case series describes three patients presented initially with red-eye and treated for conjunctivitis before the diagnosis of CCF was made.

## Case presentation

Case 1

**Figure 1:**
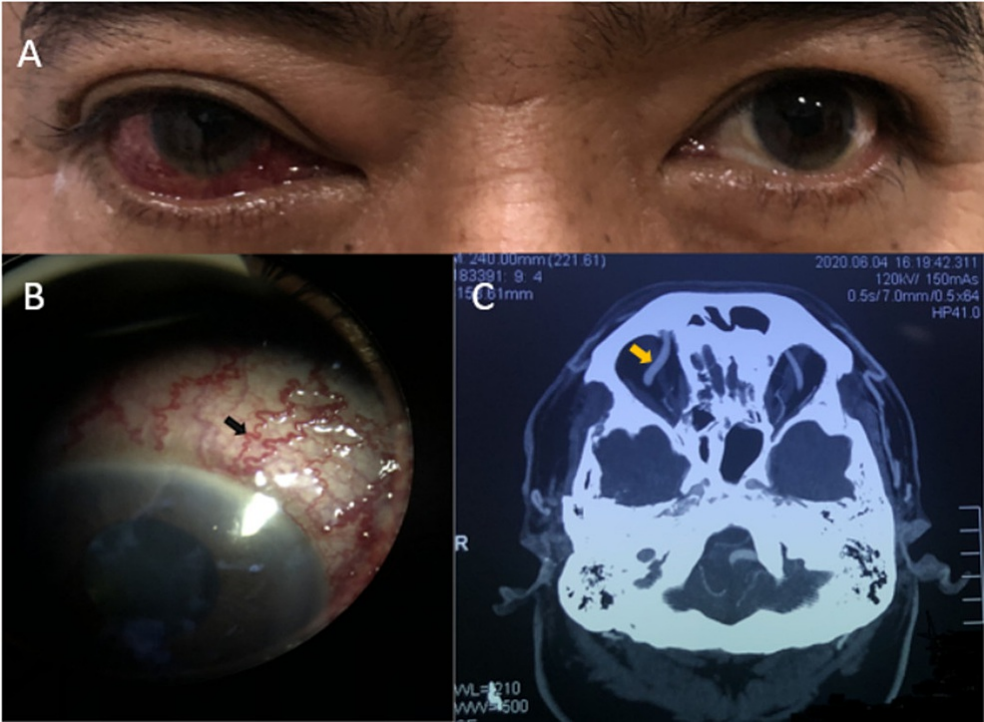
Ocular and radiological findings in Case 1. (A) Right eye redness with lid swelling. (B) Corkscrew vessels (black arrow) on slit-lamp examination. (C) Dilated right SOV (yellow arrow) in CTA.

A 56-year-old hypertensive gentleman presented with worsening right eye pain, redness, and lid swelling associated with double vision for six weeks (Figure 1A). He was initially treated as conjunctivitis at a primary care center with topical antibiotics, but then later referred to the ophthalmologist for persistent right lid eye swelling, redness, chemosis, and visual impairment. The patient recalled a past history of a motor vehicle accident in which he sustained a mild head injury with scalp laceration about 20 years ago. He had bilateral visual acuity of 6/9. His right eye was proptosed with the presence of grade 1 relative afferent pupillary defect (RAPD). It was swollen, tender with palpable thrill, and an audible bruit was present. Slit-lamp examination revealed generalized episcleral congestion with corkscrew vessels (Figure 1B) and raised intraocular pressure (IOP) of 28 mmHg. Both optic discs and posterior poles were normal. An urgent computed tomography angiography (CTA) demonstrated an engorged right superior ophthalmic vein (SOV) (Figure 1C) and bulky right CS. The left SOV was prominent with a similar contrast enhancement to the internal carotid artery. He then underwent a digital subtraction angiography (DSA) which confirmed the presence of the right direct CCF. After an urgent successful embolization, his ocular symptoms resolved fully with normalized IOP and optic nerve functions.

Case 2

**Figure 2:**
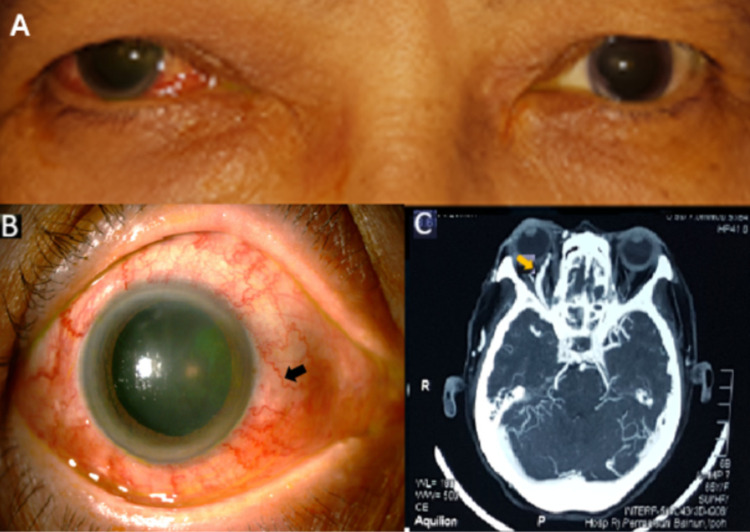
Ocular and radiological findings in Case 2. (A) Right eye redness with lid swelling. (B) Corkscrew vessels (black arrow) on slit-lamp examination. (C) Dilated right SOV (yellow arrow) in CTA.

A 65-year-old diabetic, hypertensive lady with hyperlipidemia presented with right eye blurring of vision associated with redness, lid swelling, and double vision for one month (Figure 2A). She was first treated for conjunctivitis by a private practitioner with topical antibiotics, who then referred to us for worsening ocular symptoms. Further history taking revealed a mild head trauma and facial injury in a motor vehicle accident about two months ago. A brain computed tomography (CT) scan was done and reported as normal. Her vision was 6/9 in both eyes, ocular examination showed the presence of grade one RAPD, proptosis, ophthalmoplegia, and audible bruit. Slit-lamp examination showed the presence of conjunctival chemosis and corkscrew vessels (Figure 2B) with IOP measured at 29 mmHg on the right eye. Both posterior segments of the eyes were normal. The patient underwent an urgent CTA, which showed enlarged and early filling of the right SOV (Figure 2C) with the early arterial enhancement of the right CS. The diagnosis of right direct CCF was confirmed with DSA and embolization was successfully performed to resolve the ocular sequelae.

Case 3

**Figure 3:**
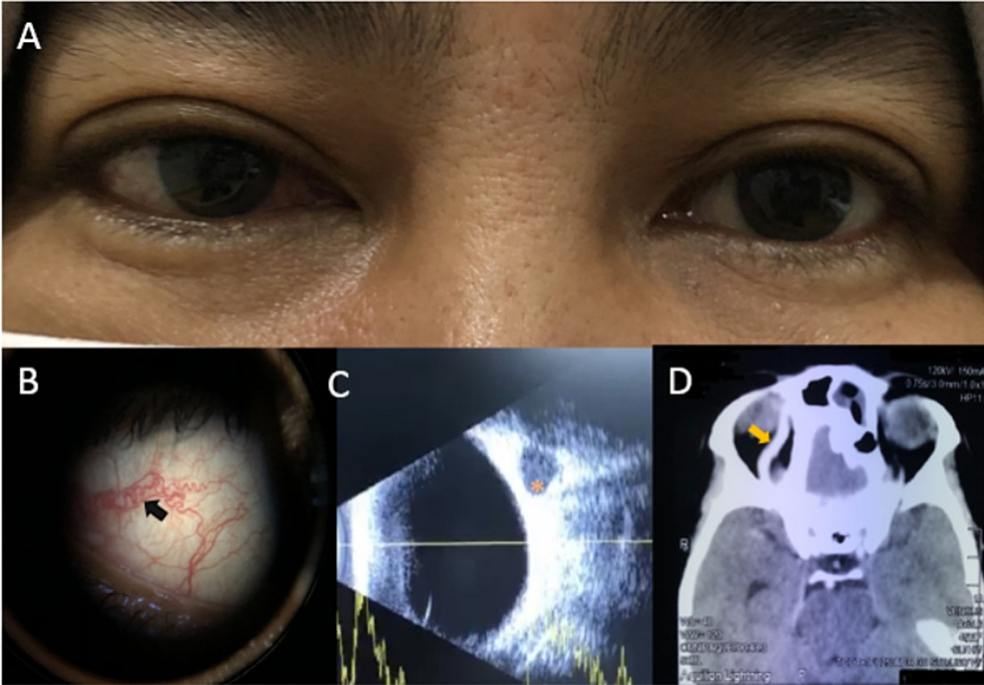
Ocular and radiological findings in Case 3. (A) Right eye redness with lid swelling. (B) Corkscrew vessels (black arrow) on slit-lamp examination. (C) Dilated right SOV (asterisk) in ultrasonography. (D) Dilated SOV (yellow arrow) in CTA.

A 49-year-old woman with underlying hypertension, was initially treated for right eye infective conjunctivitis. She was then referred for persistent and worsening eye redness for three weeks (Figure 3A). She denied any blurring of vision, lid swelling, or trauma. Her vision was 6/9 in both eyes with normal IOP. The dilated right episcleral vessels were blanched with topical phenylephrine 2.5%, hence she was treated for episcleritis with topical steroids. Her ocular condition improved after two weeks except for right eye IOP, which was raised to 24 mmHg. A topical IOP lowering agent was initiated for the working diagnosis of steroid responder. On subsequent review a week later, she was noted to have proptosis, orbital bruit, corkscrew vessels (Figure 3B) with controlled IOP and normal optic nerve functions. B-scan ultrasonography showed dilated SOV (Figure 3C) in the right eye. The patient underwent an urgent CTA that demonstrated the dilated right SOV (Figure 3D). Her DSA further confirmed the diagnosis of right indirect CCF. All the ocular features resolved following a successful embolization.

## Discussion

CCF can be classified based on etiology, rate of flow, and angiographic architecture. CCF may occur spontaneously or post-trauma. They are thought to be caused by a lesion in the cavernous wall of the internal carotid artery or one of its small branches. The majority of traumatic CCF is associated with head trauma and very few occur post-base of skull fracture [1]. Our first two patients had a history of head trauma respectively at 20 years and two months prior to the onset of ocular symptoms. Both of them sustained motor vehicle accident-related head injuries that did not require neurosurgical intervention. Eliciting previous head trauma is thus very essential, when present in a patient with a persistent red eye as it alerts the possibility of CCF.

Barrow et al. classified CCF into four subtypes based on fistula communications. Type A is a direct CCF with a high flow rate and types B, C and D are indirect CCFs with low flow rates. Type A CCF is direct communication between ICA, and CS, type B is communication between dural ICA branches, and cavernous sinus, type C is communication between dural external carotid artery branches, and cavernous sinus and type D is a communication from dural branches of ICA, and external carotid artery branches to the CS [2].

Patients with CCF may present with various ocular complaints depending on the type and severity of the vascular abnormality. They may present with classical clinical features that directly lead to the diagnosis or subtle and nonspecific signs that cause delay or misdiagnosis.

Direct CCF occur due to a sudden rise in pressure within the ICA which is commonly associated with trauma. They commonly present with orbital bruit, proptosis, chemosis abducens nerve palsy, and conjunctival injection, and venous stasis retinopathy [3]. Patients may also present with ophthalmoparesis without any congestive ocular symptoms when arterial blood is directed back into the superior petrosal sinus or inferior petrosal sinus [4]. Both of our direct CCF patients presented with these typical ocular findings that lead to a preliminary diagnosis of CCF when referred to our center. Indirect CCF usually is a low rate fistula and presents with mild orbital manifestation [5]. They may present with arterialization of the conjunctival veins, chemosis, proptosis, ophthalmoparesis, cranial bruit, retro-orbital headache, elevated IOP, and decreased visual acuity [6]. This explains the third patient in our case series who presented with mild orbital manifestations. Hence, a proper eye examination is important to elicit the presence of proptosis in red-eye cases. Clinical assessment of extraocular muscle motility has to be performed to detect any ophthalmoparesis, especially in patients with diplopia. Timely referral to the ophthalmology team for further workup is critical to prevent visual loss.

The differential diagnosis of a patient presenting with painful unilateral proptosis includes orbital cellulitis, posterior scleritis, thyroid eye disease, pseudotumor, retrobulbar hemorrhage, CS thrombosis, orbital malignancy, and vascular malformation of orbit and CS [7]. Clinical presentation of proptosis warrants further workup and imaging study.

Ultrasonography is useful to exclude posterior scleritis, extraocular muscle swelling, and retro-orbital lesions such as an intraorbital tumor. In some cases, dilated SOV can be detected through ultrasonography [5] as seen in Case 3 (Figure 3D). SOV dilatation detected in B-scan can be due to various pathologies such as CCF, SOV thrombosis, orbital hematoma, orbital tumors, or Grave’s disease [8]. CTA and magnetic resonance angiography (MRA) of the brain and orbit is performed to detect the presence of CCF. Abnormal vascular changes such as engorged venous sinuses, arterial or venous aneurysm, arterial dissection, SOV dilatation, extraocular muscle swelling, and ocular congestion can be demonstrated on CTA and MRA too [5,9]. In a series where 113 patients with radiographic evidence of dilated SOV, it was found that 44% of patients had indirect CCF and 19% had direct CCF [10]. Although CTA and MRA are non-invasive modalities that can be used in the diagnosis of CCF, DSA remains the gold standard due to its capability to localize the lesion [11].

There are different treatment options available for direct and indirect CCF. The aim of the treatment in CCF is to eliminate the fistula and maintain the ICA patency [3]. This can be achieved by co-managing with an interventional radiologist. The ICA patency can be achieved by a transarterial obliteration of the fistula with a detachable balloon, embolization with coils, or obliteration of the ipsilateral CS with embolic materials [12]. Conservative treatment can be an option in low-risk, low-flow indirect CCF [13].

Jacobs et al. reported 45% of the patients in a CCF case series were misdiagnosed by their first provider. The most common misdiagnosis noted to be infectious conjunctivitis [14]. All three patients in our series were initially treated for presumed conjunctivitis with topical antibiotics. However, due to lack of improvement and progressing clinical findings of proptosis, chemosis, and audible bruit they were later investigated for CCF. Hence, thorough history taking and ocular examination are mandatory in patients with red-eye to prevent misdiagnosis of conjunctivitis. The history-taking should include type and amount of discharge, visual changes, the severity of pain, photophobia, previous treatments, presence of allergies, and use of contact lenses. The ocular examination should include a vision test, swinging flashlight test for RAPD, external ocular examination to look for proptosis, and extraocular muscle assessment to detect the presence of restricted ocular motility or cranial nerve palsies and conjunctival arterialization, that appears as a corkscrew vessel. Serious ocular implications of a delay in diagnosis may result in visual loss, as a result of optic neuropathy, secondary glaucoma, and choroidal effusion or detachment. In severe cases, patients may develop intracerebral hemorrhages and cerebral venous infarct [15].

## Conclusions

CCF is a potential sight and life-threatening condition. Failure to recognize the different characteristics of the red-eye may result in misdiagnosis and permanent visual impairment. The clinician should have a high index of suspicion in patients with atypical presentation of red-eye and should perform a comprehensive ocular examination to identify red flags and make an early referral to an ophthalmologist for a timely diagnosis and management of CCF.

## References

[1] Helmke K, Krüger O, Laas R (1994). The direct carotid cavernous fistula: a clinical, pathoanatomical, and physical study. Acta Neurochir (Wien).

[2] Barrow DL, Spector RH, Braun IF, Landman JA, Tindall SC, Tindall GT (1985). Classification and treatment of spontaneous carotid-cavernous sinus fistulas. J Neurosurg.

[3] Lewis AI, Tomsick TA, Tew JM Jr (1995). Management of 100 consecutive direct carotid-cavernous fistulas: results of treatment with detachable balloons. Neurosurgery.

[4] Wu HC, Ro LS, Chen CJ, Chen ST, Lee TH, Chen YC, Chen CM (2006). Isolated ocular motor nerve palsy in dural carotid-cavernous sinus fistula. Eur J Neurol.

[5] de Keizer R (2003). Carotid-cavernous and orbital arteriovenous fistulas: ocular features, diagnostic and hemodynamic considerations in relation to visual impairment and morbidity. Orbit.

[6] Meyers PM, Halbach VV, Dowd CF (2002). Dural carotid cavernous fistula: definitive endovascular management and long-term follow-up. Am J Ophthalmol.

[7] Naesens R, Mestdagh C, Breemersch M (2006). Direct carotid-cavernous fistula: a case report and review of the literature. Bulletin de la Société belge d'ophtalmologie.

[8] Jørgensen JS, Guthoff R (1986). Differential diagnosis of the dilated superior ophthalmic vein by B-scan ultrasonography. Orbit.

[9] Chen CC-T, Chang PC-T, Shy C-G, Chen WS, Hung H-C (2005). CT angiography and MR angiography in the evaluation of carotid cavernous sinus fistula prior to embolization: a comparison of techniques. Am J Neuroradiol.

[10] Adam CR, Shields CL, Gutman J (2018). Dilated superior ophthalmic vein: clinical and radiographic features of 113 cases. Ophthalmic Plast Reconstr Surg.

[11] Martin S, Teo M, Bhattacharya J, Alakandy L (2013). Carotico-cavernous fistula: an educational case. Int J Surg Case Rep.

[12] Gemmete JJ, Ansari SA, Gandhi DM (2009). Endovascular techniques for treatment of carotid-cavernous fistula. J Neuroophthalmol.

[13] Viñuela F, Fox AJ, Debrun GM, Peerless SJ, Drake CG (1984). Spontaneous carotid-cavernous fistulas: clinical, radiological, and therapeutic considerations. Experience with 20 cases. J Neurosurg.

[14] Jacobs SM, Arias EJ, Derdeyn CP, Couch SM, Custer PL (2015). Carotid cavernous sinus fistulas without superior ophthalmic vein enlargement. Ophthalmic Plast Reconstr Surg.

[15] Stiebel-Kalish Stiebel-Kalish (2002). Cavernous sinus dural arteriovenous malformations: patterns of venous drainage are related to clinical signs and symptoms. Ophthalmology.

